# Post-dural Puncture Headache: A Comparison Between Median and Paramedian Approaches in Orthopedic Patients

**DOI:** 10.5812/kowsar.22287523.2159

**Published:** 2011-09-26

**Authors:** Faramarz Mosaffa, Khodamorad Karimi, Firooz Madadi, Seyyed Hasan Khoshnevis, Laleh Daftari Besheli, Alireza Eajazi

**Affiliations:** 1 Akhtar Orthopaedic Hospital, Shahid Beheshti University of Medical Sciences, Tehran, Iran

**Keywords:** Post-dural puncture headache, Spinal anesthesia, Orthopedics

## Abstract

**Background:**

Post-dural puncture headache (PDPH) is an iatrogenic complication of spinal anesthesia. Reported risk factors for PDPH include sex, age, pregnancy, needle tip shape and size, bevel orientation, approach and others. Little is known regarding the effect of different approaches on the incidence of PDPH.

**Objectives:**

In this study we aimed to compare the incidence of PDPH in the case of median and paramedian approaches in patients undergoing spinal anesthesia for orthopedic operations.

**Patients and Methods:**

Patients scheduled for orthopedic surgery under spinal anesthesia between 2007 and 2008 were studied in a double-blinded randomized controlled trial. The patients were randomized to receive spinal anesthesia by either a median (n = 75) or paramedian (n = 75) approach through a 25-gauge Crawford needle. No premedication was given, and all patients received 500 mL of normal saline intravenously and 4 mL of 0.5% isobaric Marcaine 30 minutes prior to surgery in both approaches.

**Results:**

Fifteen patients (10%) developed PDPH. There was no significant difference in the incidence of PDPH in both groups, with 7 (9.3%) patients in the median approach group versus 8 (10.7%) in the paramedian approach group developing typical PDPH (P = 0.875). However, a significant difference in PDPH incidence (P = 0.041) was observed between females (9; 16.7%) and males (6; 6.3%).

**Conclusions:**

There is no difference between median and paramedian approaches with respect to PDPH incidence; the paramedian approach is therefore recommended, especially for older patients with degenerative changes in the spine and intervertebral spaces and those who cannot take the proper position. Moreover, the rate of PDPH was found to be significantly higher in females than in males.

## 1. Background

Post-dural puncture headache (PDPH) is an iatrogenic complication of spinal anesthesia. Causes reported to influence the incidence of PDPH are sex, age, pregnancy, previous history of PDPH (1), needle tip shape (2, 3), needle size (1, 2), bevel orientation (1, 4), number of lumbar puncture (LP) attempts (1), median versus paramedian approach (5), type of local anesthetic solution (6), and clinical experience of the person operating the procedure (7). Although the loss of cerebrospinal fluid (CSF) and lowering of CSF pressure is not a controversial subject, the actual mechanism producing the headache is unclear. There are two possible explanations. First, the decrease in CSF pressure may cause traction on the pain-sensitive intracranial structures in the upright position, leading to the characteristic headache. Secondly, the loss of CSF may produce a compensatory vasodilatation (8). The incidence of PDPH was 66% in 1898 (9), which was likely due to the use of large gauge, medium bevel, cutting spinal needles. In 1956, with the introduction of 22-gauge and 24-gauge needles, the incidence was estimated to be 11% (10). Today, the use of needles such as the Sprotte and Whitacre has further reduced the incidence of PDPH, which varies with the type of procedure and patients involved. PDPH is significantly more common in young females, with the highest incidence occurring in obstetric patients (10).

There are 2 common techniques used in spinal anesthesia, median and paramedian, each of which has advantages and disadvantages. The median approach is the most common technique used, but it is technically difficult, especially in geriatric patients, because they have degenerative changes in the structural components of their spine. The paramedian approach is sometimes preferred because of faster catheter insertion (11), fewer attempts at needle insertion (12), and possibility of performing the procedure in an unflexed spinal position (13); furthermore, identification of the epidural space may be easier with the paramedian technique. This technique is also less affected by osteoarthritic changes in the elderly population; however, the oblique direction is likely to cause problems when inserting the catheter-over-needle system through the epidural needle. A paramedian approach is believed to decrease the risk of PDPH, but this has not been verified in clinical trials (5).

## 2. Objectives

In this study, we aimed to compare median and paramedian approaches with respect to the incidence of PDPH in patients undergoing spinal anesthesia for orthopedic surgery.

## 3. Patients and Methods

This double-blinded randomized controlled trial was conducted in an orthopedic center during 2007 and 2008. One hundred and fifty patients, aged 15–75 years, who were of physical status I and II according to the American Society of Anesthesiologists (ASA) classification, scheduled for pelvic or lower limb surgery under spinal anesthesia, were included in the trial. The patients were randomized to receive spinal anesthesia by either a median (Group M, n = 75) or paramedian (Group PM, n = 75) approach. The two groups were well matched with respect to weight and height (Table 1). Written informed consent was obtained from each patient and the study was approved by the Institutional Ethics Committee.

No premedication was given; 30 minutes prior to surgery, all patients received 500 mL normal saline intravenously and 4 mL 0. 5% isobaric Marcaine in both approaches. The type of approach used was blinded to the patients, surgeons, the anesthesiologist who investigated patients’ outcomes, and postoperative ward personnel. The choice of whether to use the median or paramedian approach was left to the individual anesthesiologist performing the spinal block.

The patients received the spinal anesthetic through a 25-gauge Crawford needle. The bevel of the spinal needle was directly lateral, so that the dural fibers that run longitudinally were spread rather than transected. In the median approach the dural puncture was performed in the L2-3, L3-4, or L4-5 interspaces, with the patients in the sitting position. When using the paramedian approach, patients were placed in the flexed lateral decubitus position and the spinal needle inserted in the same locations, 1 cm lateral and 1 cm caudal to the spinous process, then directed cephalad and medially at an angle of 10°-15° into the subarachnoid space.

The exclusion criteria were use of oral opioids, regular use of nonsteroidal anti-inflammatory drugs, history of allergy to any medications in the study, patient refusal, contraindication for spinal anesthesia, simultaneous general anesthesia, more than one dural puncture, alcohol or drug abuse, a history of migraine or any chronic headache preoperatively or on the morning of surgery. Starting from the first postoperative day, patients were evaluated by another anesthesiologist and asked whether they were suffering from any problems concerning the anesthesia. All reports of headache were assessed with respect to the patient’s position when the headache occurred. Only position-dependent headaches, aggravated by sitting or upright position and relieved by lying down, and headaches with bifrontal or occipital location, frequently involving the neck and upper shoulders, were regarded as PDPH. Other types of headache were considered non-specific and not PDPH.

All data are expressed as mean ± standard deviation (SD). Statistical analysis of the data was performed with SPSS 15 statistical software (Cary, NC, USA), and comparisons between the groups were done using the Chi-square test. A P < 0. 05 was considered significant.

## 4. Results

One hundred and fifty orthopedic patients underwent spinal anesthesia with a median (Group M, n = 75) or paramedian (Group PM, n = 75) approach. The preoperative characteristics of the patients are presented in Table 1. There were 48 (64%) male and 27 (36%) female in each group. The average age, 48 ± 12 years in group M and 52 ± 15 years in group PM, was not significantly different between the groups (P = 0. 52). Fifteen of the 150 patients (10%) developed PDPH. Typical PDPH was seen in 7/75 (9. 3%) patients in group M versus 8/75 (10. 7%) in group PM; no significant differences were seen between the two groups (P = 0. 875; odds ratio (OR), 0. 862; 95% confidence interval (CI), 0. 676-1. 048). With respect to the effect of gender on PDPH, the 150 study participants included 96 males and 54 females, of which PDPH occurred in 6 (6. 3%) males and 9 (16. 7%) females. Statistical analysis revealed a significant difference in PDPH incidence in females and males. (P = 0. 041; OR, 3. 0; 95% CI, 1. 242-4. 758). Fourteen patients suffering from post-dural headache were treated conservatively with bed rest, analgesics, and fluids, while 1 patient received an epidural blood patch. None of the patients encountered neurologic deficit, either transient or continuous.

**Table 1.** Patient Characteristics

	Number	Gender (M/F) ^a^	Age, y, (Mean ± SD)
Median	75	48/27	48 ± 12
Paramedian	75	48/27	52 ± 15
Total	150	96/54	-

^a^ Abbreviations: F, Female; M, male

## 5. Discussion

The results of this investigation reveal a 10% incidence of PDPH in the study population. Comparison of the 2 groups indicated that the incidences of typical PDPH were 9. 3% in the median group versus 10.7% in the paramedian group, which were not significantly different. Further, PDPH occurred in 6.3% of males and 16.7% of females; these incidence rates were significantly different between the 2 genders.

The exact mechanisms leading to PDPH are still not completely understood, although several factors, particularly the patient’s age and gender, modulate its incidence. PDPH is believed to be caused by dural leakage of CSF from the iatrogenic dural puncture following diagnostic lumbar puncture or spinal anesthesia (14, 15). The median approach involves passage of needle through the supraspinal and interspinal ligaments and the ligamentum flavum, but the paramedian approach avoids the supra- and interspinal ligaments and approaches the ligamentum flavum directly after passing through the paraspinal muscles (16). The paramedian approach appears to be an easier method due to the easier positioning of patients, especially for older patients, who have sclerosed ligaments and degenerative changes in the spine and intervertebral spaces and may have difficulty assuming the proper position for the median technique (17, 18).

A study by Haider et al. on 50 patients undergoing different elective surgeries under spinal anesthesia found a statistically significant difference in the incidence of PDPH with median and paramedian approaches. They concluded that the paramedian approach using the Quincke level needle reduces the incidence of PDPH significantly (19).

In contrast, another study by Janick et al. on 250 patients undergoing transurethral prostate surgery under spinal anesthesia reported a significantly higher rate of PDPH with the paramedian approach than with the median approach in relatively older patients, while no significant difference was observed in younger patients (5).

Our findings are different from these studies, as we observe no significant difference between the two approaches regarding the rate of PDPH. The reason could be due to the identical tearing of the longitudinal dural fibers. Alternatively, despite having a different angle, due to the cylindrical shape of the dura, the orientation of the needle insertion might be the same. The differences between our findings and those of Janick et al. might be due to differences in age groups between the two studies, as patients undergoing prostate surgery are usually older. Furthermore we excluded patients with more than one puncture.

Sex is believed to be a risk factor for the development of PDPH. Some anesthesiological studies suggest that there may be no significant difference in the incidence of PDPH between males and females (1, 4, 15). Other randomized data indicate that females may exhibit a higher incidence of PDPH compared with males (7. 4% for females vs. 3. 4% for males) (20). There may be several reasons why a higher incidence of PDPH is seen in females. It is well recognized that females have a higher incidence of certain types of headaches, such as tension and migraine headaches (21, 22). In addition, females exhibit greater sensitivity to experimentally induced pain, and demonstrate greater temporal summation of mechanically evoked pain (23-25). Finally, some data also suggest that sex hormones may influence the incidence of certain types of headaches (26, 27). In our study, the incidence of PDPH was significantly higher in females than males, consistent with the results of these previous studies.

There are several studies regarding the effect of gender, needle size and type, age, and bevel direction on the rate of PDPH (2-4, 15, 20, 23, 24) but to the best of our knowledge there have been only two studies that specifically investigate the effect of needle insertion approach on the rate of PDPH (5, 19).

We conclude that there is no difference in PDPH incidence with median versus paramedian approaches, and therefore recommend the paramedian approach, especially for older patients with degenerative changes in the spine and intervertebral spaces, and those who cannot assume the proper position for the median approach; the easier positioning would result in less pain for the patient and a higher success rate for spinal anesthesia. Moreover, we observed a significantly higher incidence of PDPH in females than in males.
